# Contamination and sample mix-up can best explain some patterns of mtDNA instabilities in buccal cells and oral squamous cell carcinoma

**DOI:** 10.1186/1471-2407-9-113

**Published:** 2009-04-16

**Authors:** Hans-Jürgen Bandelt, Antonio Salas

**Affiliations:** 1 Department of Mathematics, University of Hamburg, 20146 Hamburg, Germany; 2 Unidade de Xenética, Instituto de Medicina Legal and Departamento de Anatomía Patolóxica e Ciencias Forenses, Facultad de Medicina, Universidade de Santiago de Compostela, 15782; Galicia, Spain

## Abstract

The study of somatic DNA instabilities constitutes a debatable topic because different causes can lead to seeming DNA alteration patterns between different cells or tissues from the same individual. Carcinogenesis or the action of a particular toxic could generate such patterns, and this is in fact the *leitmotif *of a number of studies on mitochondrial DNA (mtDNA) instability. Patterns of seeming instabilities could also arise from technical errors at any stage of the analysis (DNA extraction, amplification, mutation screening/sequencing, and documentation). Specifically, inadvertent DNA contamination or sample mixing would yield mosaic variation that could be erroneously interpreted as real mutation differences (instabilities) between tissues from the same individual. From the very beginning, mtDNA studies comparing cancerous to non-cancerous tissues have suffered from such mosaic results. We demonstrate here that the phylogenetic linkage of whole arrays of mtDNA mutations provides strong evidence of artificial recombination in previous studies on buccal cells and oral squamous cell carcinoma.

## Background

Mitochondrial DNA analysis of different tissues and cells from an individual is often carried out in order to learn more about the distribution of some minor variation (heteroplasmy) of mtDNA molecules within an organism and about spontaneous somatic mutations that could play a role in carcinogenesis, in particular. Such analysis is, more often than generally believed, beset with problems related to the quality of mtDNA samples, DNA extraction, PCR and sequencing protocols, and the omnipresent risk of contamination and documentation errors [1-8]. A careful design of experiments and optimal laboratory conditions will prevent most of the potential artifacts before they occur, but there can never be a full guarantee that the performed mutation screening eventually represents authentic variation. Data analysis of the fully documented screening and sequencing results should therefore be employed *a posteriori*, by using all available database and phylogenetic information [9,10]. A previous study [11] on mtDNA alterations in oral squamous cell carcinoma and a most recent study [12] on mtDNA abnormalities in buccal cells of smokers then do not seem to be exempt from the notorious problems of sample mixing and contamination.

The rationale for an *a posteriori *analysis is that mitochondrial genomes evolve along a phylogeny (genealogical tree) and are highly polymorphic. This high polymorphism is only in part due to a minority of extreme hotspot mutations (such as polyC-tract length polymorphisms) but is mainly generated by blocks of inherited mutations that are not reshuffled by recombination as in the case of the diploid autosomal genome. Since many mutations occur only very few times in parallel along the global mtDNA phylogeny, a combination of several such inherited mutations locates a sample in the phylogeny in a unique way, rendering it extremely unlikely that a complex mutational pattern could have arisen *de novo*. This allows the researcher who has the necessary knowledge about natural mtDNA variation to question mtDNA sequencing results.

## Methods

We tab into the standard databases in the field, namely MITOMAP [13] and mtDB [14]. In addition, we perform Google searches of the kind described in [15,16]. This directly leads to entire coding-region haplotypes or control-region haplotypes stored in GenBank and discussed in the Web by commercial genetic ancestry companies or their clients.

To put the recorded mutations or haplotypes into phylogenetic context, knowledge about the continental mtDNA phylogenies is drawn from various publications [17-20]. Mutational hotspots with positional mutation rates well above the rate averaged over the entire molecule are readily identified by aggregating the macro-haplogroup trees and counting the recurrent changes at each site; see [21] and Table 4 of [22]. Numbering of mutations and polymorphisms along the mtDNA genome are referred to the revised Cambridge Reference Sequence, rCRS [23].

## Results

### Oral squamous cell carcinoma

Prior et al. [11] extracted mtDNA from 30 paired samples of tumour and non-tumour tissue, which was analyzed for contrasting variation within the two short fragments 4527–4954 and 30–407. These ranges constitute the putatively readable parts of the amplicons, excluding the primer locations, although it is not clear from their article whether the entire ranges and both strands were in fact analyzed and well readable from start to end. Note that the analysis of both strands and re-sequencing of the same DNA extracts and amplicons do not prevent artificial recombination due to e.g. sample mix-up; only separate and individual extractions from the same individual could provide the minimum guaranties [4]. The observed paired mtDNA sequences were unfortunately not reported in Prior et al. [11], so that no *a posteriori *check is possible with respect to the potential completeness of the mutation profiles compared to the worldwide database of published mtDNA sequences. The information that is given in the two tables concerns only the nucleotide differences between the two sequences from each pair of analyzed tissues.

**Figure 1:**
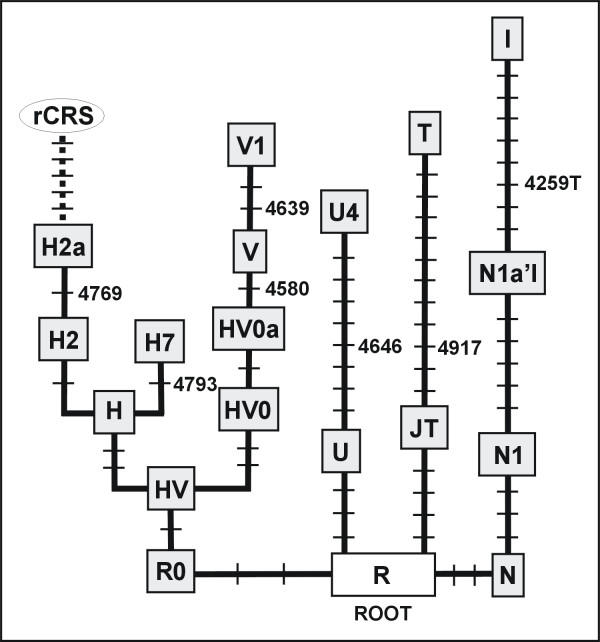
**View on the West Eurasian mtDNA phylogeny through the narrow window 4527–4954**. Boxed letters designate haplogroups and rCRS (revised Cambridge Reference Sequence) and α-γ are specific lineages. Each bar indicates a mutation, which is numbered when it occurred within the window. All mutations are transitions unless suffixed by a letter in case of a transversion (to T or C) or a deletion (d). Nomenclature of haplogroup H2 and H2a is as in [20].

The first table then provides the contrasting variation for the portion 4527–4954 of the ND2 gene. Assuming that the patients had West Eurasian matrilineal ancestries, we first take a look at the basal part of the corresponding mtDNA phylogeny by focussing on the classified parts as reflected in Figure 1 of Palanichamy et al. [17]. Then only seven mutations within that short fragment highlight haplogroups that were well described before 2005; see our Figure 1. Alternatively, one can search the mtDB database for variation within this fragment and consider, say, the top twelve most frequently recorded variants. Among those, there are seven that predominantly are of East Asian provenance and thus not relevant here. Then the five remaining frequent variants are A4529T, G4580A, A4769G, A4793G, and A4917G, which are – not incidentally – all signifying basal haplogroups in Europe (Figure 1). The fact that these variants are frequently stored in the database does not mean that they are mutational hotspots, but rather reflects the inheritance of the corresponding mutational variant in European matrilines. For instance, no position in the region 4527–4954 would qualify as a mutational hotspot with more than three mutations in the mtDNA tree of Kivisild et al. [21].

The nucleotide variant located in this DNA fragment that is by far most frequently found in Europe is the nucleotide inherited from the transition A4917G characterizing haplogroup T, which has frequency ~10% across Europe. Next in frequency (~5%) comes G4580A in West Europe, characteristic of haplogroup V. Now, if one would randomly permute paired identical sequences, that is, deliberately generate mosaic assignments, then the typical sequence contrast would involve position 4917 and to a lesser extent 4580 and finally positions 4529, 4769, and 4793. Now, the variation recorded in Table 1 of [11] mirrors exactly such hypothetical sample mixing: four events at 4917, which could reflect perfect sample crossover (since two changes are A to G and two are G to A), whereas one event is at 4580 and another one at 4769! Thus, it is very unlikely that the recorded haplotypic variation presents authentic somatic variation, which would be expected to hit positions from the entire fragment randomly, and not just affect those few that signify major European haplogroups.

The fragment 30–407, which encompasses the so-called second hypervariable segment of the control region, harbours a number of extreme mutational hotspots. The top-most changes are the transitions at positions 146, 150, 152, 195, and the length polymorphism of the C stretch preceding position 310, which is often found in heteroplasmic state in individuals, healthy or not. This entails that transitional changes at positions 146, 150, 152, and 195 can be found in almost all combinations (haplotypes) among samples from the general population. In contrast, relatively rare changes are, for instance, the transversion C186A and the transitions C186T, A240G, C285T, and C295T, where the latter two variants are excellent markers for haplogroups U1 and J, respectively.

The salient feature of the changes recorded in Table 2 of Prior et al. [11] is that whole arrays of mutations (such as the motif T146C-T152C-C186A), involving several hypervariable as well as single stable sites, switch back and forth. Such a concerted mutational process has never been observed in mtDNA population studies other than in cases of artificial recombination of amplicons [6,24]. One can, for instance, interpret the mtDNA alterations in Patient 10 as the result of sample mix between a genuine haplogroup V mtDNA (testified by G4580A, and carrying a rare private change A240G) and a haplogroup J1c sequence (indicated by G228A and C295T).

We conclude that virtually all the mtDNA alterations recorded in the study of Prior et al. [11] can be perfectly explained by sample mixing and contamination. The observed pattern itself is incompatible with features of a natural mutational process that would never change linked complex arrays of point mutations back and forth.

### Buccal cells

The study by Tan et al. [12] reported numerous mtDNA alterations in buccal cells from smokers versus non-smokers. The tables in this paper display only the cumulated differences between mtDNAs in lymphocytes and in buccal cells within the cohort of smokers and that of non-smokers, respectively, but do not provide the information about the set of alterations (haplotypes) in every single individual. Such haplotype information is usually necessary for critical reading and interpretation of the experimental results since it directly hints at potential artifacts. In this case, however, the surprisingly large number of seeming homoplasmic somatic changes, although just cumulatively listed in their Table 3, is quite telling because a number of mutations are known to occur together on pathways of the mtDNA phylogeny.

Specifically, the nine mutations at sites 10086, 10373, 10398, 13105, 15824, 15944del, 16223, 16278, and 16362 unambiguously determine a haplogroup L3b1 lineage α, the seven mutations at 10115, 10530, 10398, 13590, 13650, 16223, and 16278 signify a particular mtDNA lineage β belonging to haplogroup L2c [21], and the five mutations at 4529, 10034, 10238, 10398, and 16223 indicate a lineage γ of haplogroup I status. The presence of these lineages in the samples analyzed by Tan et al. [12] (see their Table 3) gives a strong signature of contamination or sample mix-up. Indeed, among 2704 entire coding region sequences stored in the mtDB [14], T10115C exactly identifies the 61 haplogroup L2 sequences, A4529T the 33 haplogroup I sequences, and A15824G the 17 haplogroup L3b sequences – without exception. These mutations are thus excellent haplogroup markers on their own. The fact that even 3–5 additional coding region mutations (plus further mutations from the more variable control region) come in support of the respective haplogroup profile renders the haplogroup signature bullet-proof.

**Figure 2:**
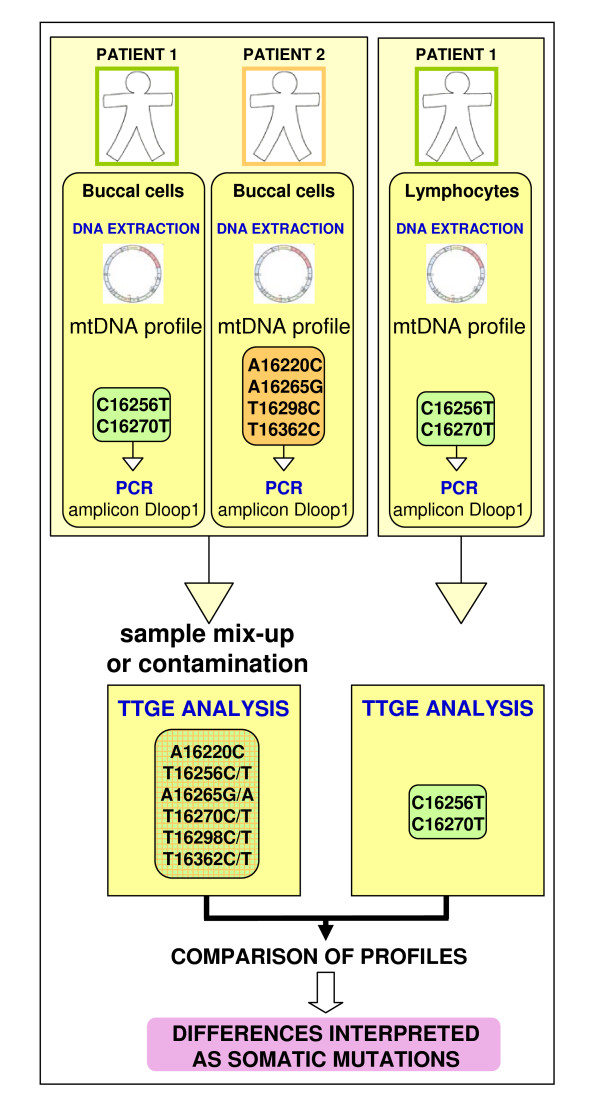
**Diagram illustrating a sample mix-up event that would explain part of the artefactual results from Tan et al. **[12].

In addition, one could further postulate the presence of a particular haplogroup T2b lineage δ bearing the four mutations at 930, 9947, 13563, and 16294 [25], although none of the mutations alone would be characteristic of this lineage. In fact, G9947A would also enter the motif of haplogroup F3b and C13653T that of haplogroup G2. With respect to the amplicon range "Dloop1" (16100–16544, including primer locations) [26], the four mutations at A16220C, A16265G, T16298C, and T16362C point to a specific lineage ε belonging to haplogroup F3b that thrives in Taiwan [27], whereas the mutation pair for C16256T, C16270T testifies to a frequent European lineage ζ from haplogroup U5a. Since the former mutations are reflected in the mtDNA from buccal cells and the latter mutations in the mtDNA from lymphocytes, the presence of these six mutations could be explained by a single sample-mixing event in the laboratory: contamination of the buccal sample ζ with the buccal sample ε at the amplification step for the "Dloop1" fragment. The cartoon of Figure 2 thus reconstructs a possible scenario that could explain this contamination event.

Given the amplicon ranges [26] and the frequencies of the changes observed, one can attempt to provide a most parsimonious explanation of the mutational pattern. Besides the "Dloop1" amplicon, the long amplicon "GR" (9827–10629) covers several mutations from lineages α, β, γ, and δ. The amplicon "ND5.2" (12949–13738) covers mutations from lineages α, β, and δ. Three further amplicons then cover 930, 4529, 15884 and 15944del, respectively. This means that a few amplicons have been drawn from the wrong lineages in at least four cases (assuming the simplest scenario that the order of the nucleotides at 10034 and 13105 should rather be reversed in that table). The precise recombination events are impossible to reconstruct unambiguously in the absence of haplotype information but the signal for artificial recombination is beyond doubt. Moreover, we cannot assume that all distinguishing mutations were actually recorded, since it has been stated that TTGE can detect many but not all mutations [28]. The expected percentage of missed mutations in the case of mtDNA does not seem to have been determined systematically. Therefore the full amount of contamination and falsely assigned amplicons may be larger here, especially with amplicons that are distinguished only by a single mutation.

**Figure 3:**
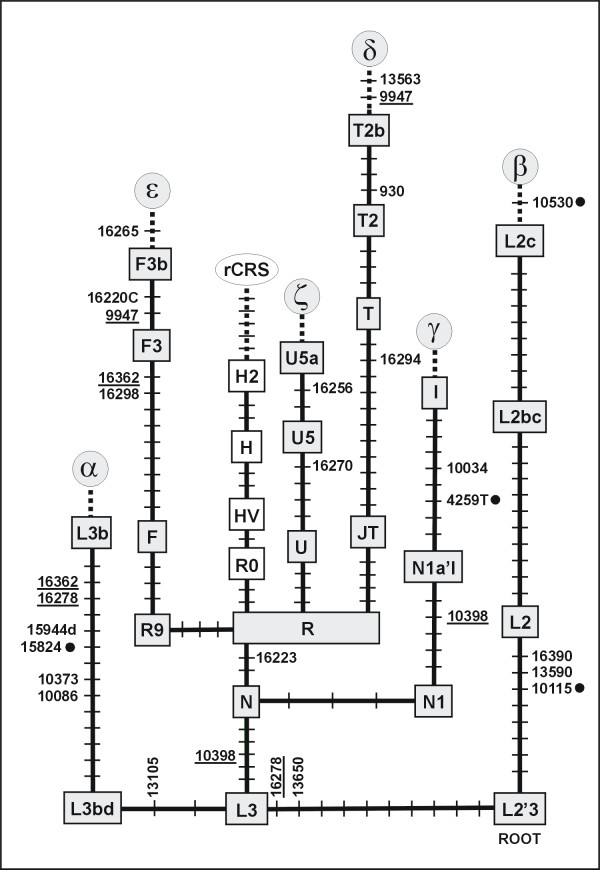
**Mutations listed by Tan et al. **[12]** that jointly highlight well-known pathways in the mtDNA phylogeny**. For symbols, see legend to Figure 1. Recurrent mutations are underlined.

The pattern of lineage mixing that nonetheless emerges from our Figure 3 gets indirect support from Table 1 of Tan et al. [12] in that the most dramatic amount of altogether 40 differences is clustered in just 8 of the 42 smokers. This, however, does not imply that 1–3 mtDNA alterations between matched samples would not be suspicious. For instance, the alterations other than A2444C recorded for the non-smokers could all signify the contrast between haplogroup L2 and other lineages. The number of wrongly assigned amplicons in the non-smoker group likely did not exceed one per individual.

An artifactual status of the findings in Tan et al. [12] is also reflected by the extremely uneven distribution of seeming mtDNA alterations across the different amplicons, which is a typical feature of mixing up different mtDNA lineages at some but not all amplification steps. Another amplicon-specific feature is the seeming length variation of homopolymeric A or C tracts, which was recorded only for the amplicon "SD" (7234–7921), although several other notorious tracts exist elsewhere in the molecule.

## Conclusion

Neither of the two studies published in the journal Carcinogenesis which we have reassessed here can provide any convincing indication of an increased amount of somatic point mutations in smokers or cancerous tissues, since the reported variation shows the typical imprint of mosaicism of amplicons caused by inadvertent contamination and sample confusion

Once a problematic mtDNA study is published, the authors of the study and the reader are in very unequal positions: Whereas the former possess the samples and know all the data on which the article was built, the unconvinced reader who has to make do with the meagre portion of the data that is revealed in the article can only base his arguments on reconstructed haplotypic data (as in the two cases that we have re-examined here).

Artifactual patterns differ from the natural ones in that they usually mirror basal parts of the mtDNA phylogeny whence they can easily be detected by comparison with published mtDNA classification trees [29]. In contrast, the natural instabilities are expected to result from a stochastic mutation process that randomly hits nucleotide positions along the mtDNA molecule and does not just run down and climb up the mtDNA phylogeny. On the other hand, one cannot a priori preclude the possibility that some damage may exist in the form of an elevated amount of large deletions in mtDNAs of exposed cells. However, results would always come under suspicion of experimental error and thus warrant revision whenever single exposed tissues or tumoral samples showed an atypical pattern of (seeming) instabilities. It is then not only the kind of mutation involved (whether highly or lowly mutable), but also the combination of these mutations in haplotypes and, moreover, the multiple appearance of such 'yin-yang' haplotypes (*sensu *e.g. [30,31]) in several individuals, as in the case of Prior et al. [11], that render the results highly dubious.

A study on brain tumors that compared matched cancerous/non-cancerous tissues and was executed under forensic sequencing conditions [3,32] did not find any remarkable mtDNA alterations: the vast majority of changes in the two hypervariable segments of the control region concerned the homopolymeric C tract, and in no case among 69 patients was more than one substitution found [33]. A similar pattern can also be seen in the study on esophageal cancer by Tan et al. [34] (which was duplicated from [35]): in the entire mtDNA of 20 matched pairs of samples (analyzed with TTGE) there were only five singular changes found other than instabilities of that homopolymeric C tract.

In order to minimize the risk of contamination or sample confusion going undetected it is advisable to use at least twice the number (32) of primer pairs for amplification that was employed by Tan et al. [12]. Every nucleotide position should thereby be covered by at least two distinct amplicons, a requirement that e.g. forensic scientists should ideally meet [36]. This would also enhance the chances for TTGE to detect a mutation prior to sequencing. Further note that a good quality electropherogram is no guarantee of an authentic result: when samples are mixed up initially, one can observe perfect sequencing patterns (bands) just showing (seeming) heteroplasmies at those unmatched positions between the two mixed haplotypes. Finally, any discrepancy between the results for two amplicons that indicated a mutated position in one but not the other amplicon would definitely need re-sequencing. In case there are no mutations in the overlap regions of an amplicon with its two neighbouring amplicons, then a strict association of the yin-yang pattern with different amplicons gives a strong signal for sample crossover [29]. Since samples may arrive in the laboratory in problematic state [1], it would be wise to follow some guidelines usually exercised in the field of ancient DNA; in particular, one should perform a critical consideration of all available information and answer the question "Is there any reason to not believe the results or conclusions?" [37].

### Response

By Dr Paul Lewis, Dr Paul Griffiths, Dr Sarah Prior

Institute of Life Science, School of Medicine, Swansea University, Swansea, UK

In their paper 'Contamination and sample mix-up can best explain some patterns of mtDNA instabilities in buccal cells and oral squamous cell carcinoma' Bandelt and Salas have assessed the authenticity of somatic mtDNA mutations observed in oral squamous cell carcinoma (OSCC) reported by Prior et al [11]. The phylogenetic approach used by Bandelt and Salas utilizes publicly available mtDNA sequence data to evaluate whether reported somatic mutations are nothing more than polymorphisms occurring between different mtDNA haplotypes that co-exist due to sample mix-up. In previous publications Bandelt and Salas have quite rightly called into question the reporting and authenticity of mtDNA mutations and even suggested 'rules' on error detection and quality control [4,38-40]. We strongly applaud these efforts and thoroughly agree that journals should employ strict rules on provision of sequence data by submitting authors as well as guidelines for journal reviewers.

Bandelt and Salas have demonstrated use of their phylogenetic approach in a previous study of mtDNA mutations in different tumour types [4]. In that *a posteriori *study of sequence data derived from many samples they were able to highlight a number of mutations that were not somatic as originally reported, but simply polymorphisms between haplotypes occurring in the sample after a likely sample mix-up. We were mindful of the guidelines and problems previously highlighted by Bandelt and Salas when reporting the results from our study on mtDNA mutations in OSCC. PCR products were sequenced multiple times (in both directions) and only consistent mutations described. When we submitted our manuscript to Carcinogenesis one of the reviewers instructed that, before mutations could be authenticated, PCR products would have to be generated using high fidelity PCR to minimize the likelihood of error. Thus, despite having already replicated PCR and sequencing a number of times for each sample we had to confirm our results by repeating this process once more and further submit this data. The second reviewer demanded that we 'repeat PCR and sequencing of all positive samples independently to exclude the possibility of contamination'. Thus, the mutations we reported were done so with a high degree of confidence and we can clearly state that Carcinogenesis demonstrated a high degree of caution prior to acceptance of our results. Our sequence data were available at all times during the review process.

In our study of OSCC cases six patients (out of thirty) individually showed a single base substitution between tumour and normal tissue within a 473 bp region of the ND2 gene. These mutations were observed at three nucleotides: 4580 (1 patient), 4769 (1 patient) and 4197 (4 patients). The frequencies of these mutations and pattern, according to Bandelt and Salas, reflect a hypothetical sample mixing. They conclude that 'it is very unlikely that the recorded haplotypic variation presents authentic somatic variation, which would be expected to hit positions from the entire fragment randomly'. This is a conclusion drawn on only a single nucleotide per sample and an assumption that the nucleotide target specificity of mutagens is surprisingly random. Bandelt and Salas go on to conclude that somatic mutations observed in the D-Loop of OSCC in our study are also due to sample mixing and that mutation patterns observed at nucleotides 146, 152 and 186 are incompatible with features of a 'natural mutational process'. We are intrigued as to what the natural mutational process may be in oral epithelial cells of cigarette smokers. They go on to state that 'the natural instabilities are expected to result from a stochastic mutation process that randomly hits nucleotide positions along the mtDNA molecule...'. If we adopt this rationale then we will always discard those legitimate somatic mutations that occur at polymorphic sites (it is ironic too that polymorphic sites are actually markers of mutation events sometime in the past).

We are surprised that we are expected to believe that mtDNA somatic mutations would be found at random positions in the genome particularly when the genome of a smoker is exposed to constituents of cigarette smoke. Cigarette smoke contains over 4000 chemicals [41] many of which are known to be mutagenic. There are literally thousands of publications for mutagenicity assays highlighting the fact that mutagens cause base substitutions which do not appear randomly in a target gene but demonstrate sequence specificity. As an example we consider the polycyclic aromatic hydrocarbon (PAH) carcinogen (±) anti-7β,8-dihydroxy-9,10-epoxy-7,8,9,10-tetrahydrobenzo [a]pyrene (BPDE) found in cigarette smoke that preferentially targets CG dinucleotides (unmethylated and methylated) at a high rate in the supF gene using the supF mutational assay [42]. There is also a strong correlation between BPDE adduct sites and mutation hotspots in the TP53 gene in lung cancer [43]. A search of the literature would reveal that many other mutagens specifically display other types of sequence context. The exposure of mtDNA to mutagens from cigarette smoke could thus result in a non-random mutation spectrum. The non-random pattern of mutations occurring in tandem at nucleotides 146 and 152 in a number of samples from our study is quite plausible. Tandem mutation events are common in mutagen-induced mutation spectra from mutagenicity assays. Interestingly, tandem mutations have been observed at nucleotides 146 and 152 when comparing a lung cancer cell line with a matched B-lymphoblastoid cell line [40].

Given that we suggested that mutation hotspots in our study were occurring in the OSCC tumours we can understand why Bandelt and Salas raised concerns about a 'switch back and forth' for mutations at 146 and 152. However, it is unknown as to whether mutations observed by us at 146 and 152 in OSCC actually occurred in the tumour or non-tumour tissue for each patient examined. Thus, it is unknown whether a switching in this sense has actually occurred. This could be confirmed by sequencing PCR products from a third tissue per case. In all probability we were wrong to conclude that the 146 and 152 mutations are potential biomarkers for OSCC but they may well prove to be useful biomarkers for smoking related DNA damage.

In the reassessment of our data using their phylogenetic linkage approach Bandelt and Salas state that they provide a 'strong evidence of artificial recombination' and that virtually all the mtDNA alterations can be explained by sample mixing and contamination. It is interesting that the logic applied by Bandelt and Salas means that any single somatic mutation occurring at a nucleotide that is polymorphic between different haplotypes can simply be explained by sample mixing. In other words, by just applying the phylogenetic approach to studies involving few mtDNA mutations in such a small region of genome, you can never provide proof of contamination but just make inference. We fully respect the opinions of both authors and support their quest for accurate procedure and reporting. However, to make accusations in the literature of results generated due to contamination without presenting any conclusive evidence is unwarranted and thankfully not acceptable by most journals. We question how anybody can draw such conclusions based on tiny pieces of data lacking empirical evidence and statistical support and, seemingly, a lack of understanding of mutagen associated sequence context. Reviewers at journals should insist on reasonable use of the phylogenetic approach applied by Bandelt and Salas in addition to the rules that these authors suggest.

## Abbreviations

mtDNA: mitochondrial DNA; rCRS: revised Cambridge Reference Sequence.

## Competing interests

The authors declare that they have no competing interests.

## Authors' contributions

H-JB and AS designed the study, analyzed the data, and wrote the paper.

## Pre-publication history

The pre-publication history for this paper can be accessed here:

http://www.biomedcentral.com/1471-2407/9/113/prepub
